# Abortion with Placenta Prœvia and Hydrops Amnii

**Published:** 1855-08

**Authors:** D. W. Hershey

**Affiliations:** Freeport, Ill.


### ART. II.—*Abortion with Placenta Prævia and Hydrops Amnii.*

By D. W. Hershey, M. D., Freeport, Ill.

Early in the morning of the 15th of June, I was called to the village of Cedarville, about six miles distant, to consult with Dr. Bucher, of that place. Having arrived there, the following history of the case was detailed by him:

On the preceding evening the patient, Mrs. B., while engaged in some domestic duties, was seized with severe labor pains: she was but in her fifth month of gestation. Dr. B. was immediately called in, and made every effort to quiet the pains and prevent the expulsion of the uterine contents.

Notwithstanding all his efforts, the pains continued, the sac presented and protruded of an enormous size. It soon ruptured, and was followed by the discharge of an unusual quantity of fluid: Dr. B. thinks as much as two gallons. This discharge was succeeded by several forcible contractions, attended by as many copious discharges of blood.

It was at this juncture Dr. B. dispatched a messenger for aid. In the mean time, by my arrival, he had succeeded, by the ordinary appliances, in arresting the hæmorrhage.

I found the patient nearly exsanguine; pulse 120; surface cool. A vaginal examination revealed the fœtus in the left cephalo illiac position, the arm presenting and extruded. The placenta was detected slightly encroaching upon the os. From the extreme exhaustion of the patient it was thought advisable to resort to stimulants. Wine was administered at intervals, and by 11 o'clock, A. M., reäction had commenced. Pulse 80, and full; countenance flushed, and labor recommenced. The uterus had contracted down firmly upon its contents, the os grasping the arm so closely that it was impossible to introduce a finger.

Recourse was now had to the sedative virtues of morphia, and the patient having been brought fully under its influence, some little advantage was gained. The os yielded sufficiently to enable me to tilt up the body of the fœtus, so that the head presented and delivery was soon accomplished.

There are several interesting points in this case: the large amount of liquor amnii, it being the probable cause of the supervention of labor, and the consequent hæmorrhage resulting from the position of the placenta.

Should the above case be of sufficient interest to warrant its publication, it is at your service.

